# A benchmark study on error-correction by read-pairing and tag-clustering in amplicon-based deep sequencing

**DOI:** 10.1186/s12864-016-2388-9

**Published:** 2016-02-12

**Authors:** Tian-Hao Zhang, Nicholas C. Wu, Ren Sun

**Affiliations:** Department of Molecular and Medical Pharmacology, David Geffen School of Medicine, University of California, Los Angeles, 90095 CA USA; School of Life Science, Fudan University, Shanghai, 200433 China; Molecular Biology Institute, University of California, Los Angeles, 90095 CA USA; Department of Integrative Structural and Computational Biology, The Scripps Research Institute, La Jolla, 92037 CA USA

**Keywords:** Deep sequencing, Amplicon sequencing, Error-correction, Tag-clustering, Read-pairing, Error rate

## Abstract

**Background:**

The high error rate of next generation sequencing (NGS) restricts some of its applications, such as monitoring virus mutations and detecting rare mutations in tumors. There are two commonly employed sequencing library preparation strategies to improve sequencing accuracy by correcting sequencing errors: read-pairing method and tag-clustering method (i.e. primer ID or UID). Here, we constructed a homogeneous library from a single clone, and compared the variant calling accuracy of these error-correction methods.

**Result:**

We comprehensively described the strengths and pitfalls of these methods. We found that both read-pairing and tag-clustering methods significantly decreased sequencing error rate. While the read-pairing method was more effective than the tag-clustering method at correcting insertion and deletion errors, it was not as effective as the tag-clustering method at correcting substitution errors. In addition, we observed that when the read quality was poor, the tag-clustering method led to huge coverage loss. We also tested the effect of applying quality score filtering to the error-correction methods and demonstrated that quality score filtering was able to impose a minor, yet statistically significant improvement to the error-correction methods tested in this study.

**Conclusion:**

Our study provides a benchmark for researchers to select suitable error-correction methods based on the goal of the experiment by balancing the trade-off between sequencing cost (i.e. sequencing coverage requirement) and detection sensitivity.

**Electronic supplementary material:**

The online version of this article (doi:10.1186/s12864-016-2388-9) contains supplementary material, which is available to authorized users.

## Background

Next-generation sequencing is being widely used in biomedical research. Several sequencing technologies, such as chained ligation (SOLiD), pyrosequencing (454), reversible dye (Illumina), fluorescent nucleotides (PacBio), and ion semiconductor (Ion Torrent) have been developed and commercialized. While different technologies have their own features (e.g. long read-length for PacBio and high output for Illumina), high sequencing error rate is a common problem for all existing next generation sequencing platforms. The high error rate significantly impedes the application of these technologies to detect rare variants in genetically heterogeneous populations.

To resolve the problems associated with the high error rate, experimental methods have been developed for distinguishing real mutations from sequencing errors. One such method is to take advantage of the paired-end feature of Illumina sequencing by removing the inconsistent forward and reverse read pairs [1–5]. Another common approach is to use nucleotide tags [6–12]. Although variations of sequencing library prepration method using nucleotide tags have been proposed, the underlying philosophy is the same. Briefly, a highly heterogeneous pool of random oligonucleotides (also known as tags or Primer IDs) is assigned to the individual nucleic acid molecules to label the original template copy. Subsequently, the same tag would be observed in different reads. This can be considered as resampling of the same original DNA template. By comparing the sequence reads that share the same tag, a corrected consensus sequence can be generated, and stochastic sequencing errors can be distinguished from real mutations. Recently, another innovative approach, known as circle sequencing [13], has been developed. With a similar design to tag-clustering methods, circle sequencing allows each DNA template to be read multiple times on a single read. These sequencing error-correction methods have been successfully applied to detect rare mutations in heterogeneous cancer tissues [14], mixed microbe populations [15], and viral quasispecies [10].

In this study, a highly uniform plasmid template from a single bacteria clone was sequenced. We applied the read-pairing correction method, as well as tag-clustering correction method to the same template. We systematically compared the error profiles and sequencing coverage of different methods to describe the pros and cons of each strategy.

## Results

### Experimental design

**Fig. 1:**
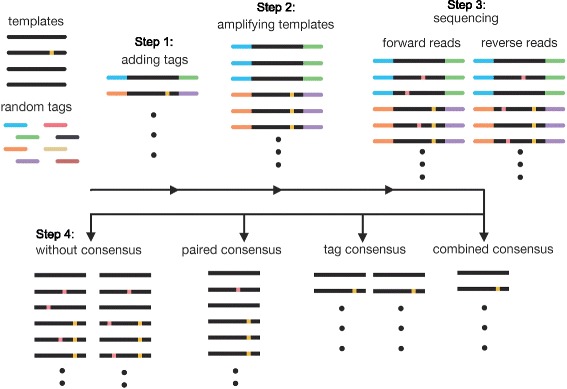
Schematic representation of the experimental design. To compare the efficiency of different error-correction methods, we generated the sequencing library in the following steps. Step 1: Linking tags to the templates. Step 2: Amplifying templates with paired end sequencing adapter. Step 3: Sequencing the library on Illumina Hiseq platform. After sequencing, we compared the efficiency of different error-correction methods. Paired-end consensus was to filter out the pairs of reads that were not identical. Tag consensus was to filter out groups of reads that were with same tags but not identical. Combined consensus used both methods for filtering. The real low frequency variants are indicated as yellow dots. And the sequencing errors are indicated as pink dots

To compare the efficiency of different error-correction methods, the sequencing library was prepared from a clonal plasmid carrying the protein G antibody interacting domain (Fig. 1). An 88 bp region of this domain was amplified through PCR. The sequence is shown in Additional file 1: Figure S1. The length of the target region in this study was similar to the read-length being used in amplicon-based deep sequencing cancer studies [16, 17]. The target region contained 54.5 % GCs. In comparison, the average GC content of human genes ranges from 34 % to 66 % [18]. Therefore, the properties of the target region in this study resembled that of the sequences of interest in other applications.

The target region was first amplified by PCR. A tag, comprising eight random nucleotides “N”, was included in both forward and reverse primers. Thus, a total of 16 random nucleotides were present in the resultant PCR product. The complexity of the tags was ∼ 4×10^9^ per sample. Around 6×10^6^ tagged molecules were then amplified to generate identical copies of each tagged molecule. The product from this second PCR was subjected to deep sequencing on the Illumina HiSeq 2500 platform. In this study, two technical replicates from the same clone were included. We were expecting ∼5 copies per tagged molecule to be sequenced, with ∼30 million sequencing reads in total. This experimental design allowed us to perform two independent error-correction approaches, namely read-pairing consensus and tag-clustering consensus. Read-pairing consensus, which was based on the sequence identities of the forward and reverse reads, was used to filter out read pairs that were unmatched. Tag-clustering consensus was 1) to group the reads by the tag sequence, and 2) to filter out groups that carried reads with different sequence identities. Based on these two error-correction approaches, we compared the results from four types of analyses: Scheme 1: Raw reads; Scheme 2: Read-pairing consensus; Scheme 3: Tag-clustering consensus; Scheme 4: Combined consensus (read-pairing consensus, followed with tag-clustering consensus).

### Error rate profiling

In this study, sequencing errors were categorized into four types namely transition (A$\leftrightarrow $G and C$\leftrightarrow $T), transversion (A$\leftrightarrow $C, A$\leftrightarrow $T, G$\leftrightarrow $C, and G$\leftrightarrow $T), insertion and deletion.

**Fig. 2:**
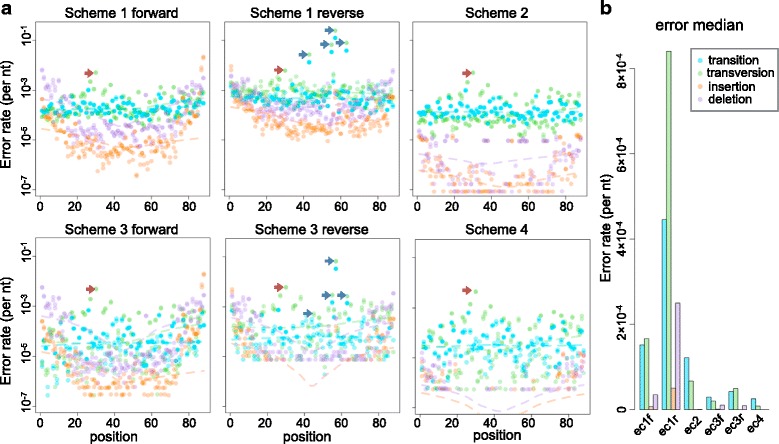
Error rates in different error-correction methods. **a** Detailed profiling of error rate on every nucleotides. Every dot represents the observed error rate on a certain nucleotide. Blue, green, orange and purple represents transition, transversion, insertion and deletion, respectively. The dashed lines represent the value of local regression. Blue arrows indicate some high rate errors. Red arrows indicate a highly possible real mutation. Two technical replicates are plotted on the same subgraph. **b** Barplot of medians of different error-correction schemes. The labels, ec1f, ec1r, ec2, ec3f, ec3r, and ec4 represent Scheme 1 forward reads, Scheme 1 reverse reads, Scheme 2, Scheme 3 forward reads, Scheme 3 reverse read, Scheme 4, respectively

In the raw sequencing data, all four error types were identified. They distributed with a peak at 10 ^−4^ per nt and a long tail to 10 ^−2^ per nt (Fig. 2a, Scheme 1 forward and reverse). The error rate was not normally distributed (Additional file 2: Figure S2, *p* <2.2×10^−16^, Shapiro-Wilk normality test). The transition rate had a median of 3.3 × 10^−4^ per nt and a mean of 1.5 × 10^−3^ per nt. The transversion rate had a median of 5.7 × 10^−4^ per nt and a mean of 3.1 × 10^−3^ per nt, which was ∼2-fold higher than transition rate. The rates of insertion and deletion errors were not normally distributed either. The rates of insertions and deletions were 10-fold lower than that of substitutions (i.e. transition and transversion), confirming that the insertion and deletion errors in Illumina platform were relatively low [19]. The insertion rate had a median of 3.2×10^−5^ per nt and a mean of 2.9×10^−4^ per nt, while the deletion rate had a median of 1.3×10^−4^ pert nt and a mean of 5.3 ×10^−4^ per nt.

All error-correction schemes improved the sequencing results significantly. But different schemes showed different advantages for correcting different error types (Fig. 2a). Read-pairing consensus (Scheme 2) significantly reduced insertion and deletion rates by ∼100-fold (*p*= 9.6 ×10^−60^, Wilcoxon signed-rank test). In contrast, transition and transversion rates were only reduced by no more than 10-fold (*p*= 2.0 × 10^−59^, Wilcoxon signed-rank test). Tag-clustering consensus (Scheme 3) reduced substitution error rates ∼20 fold (*p*= 3.9 × 10^−58^, Wilcoxon signed-rank test), but the decrease in insertion and deletion rates was only significant at the middle region of the sequencing reads (*p*= 9.6 ×10^−60^, Wilcoxon signed-rank test).

Read-pairing consensus showed significantly lower insertion and deletion rates than tag-clustering consensus (*p*= 8.0 × 10^−53^, Wilcoxon signed-rank test), while transition and transversion rates were lower in tag-clustering consensus than that in read-pairing consensus (*p*= 2.4 × 10^−12^, Wilcoxon signed-rank test). Combined consensus performed the best for both substitution rates (*p* = 1.5 × 10^−38^, Wilcoxon signed-rank test) and insertion and deletion rates (*p* = 2.9 × 10^−25^, Wilcoxon signed-rank test). The medians for all four categories of errors in different analysis scheme were shown in Fig. 2b. In conclusion, the tag-clustering correction method was very effective for substitution errors, but not for insertion and deletion errors. In contrast, the read-pairing method was very effective for insertion and deletion errors, but not for substitution errors.

In the unfiltered dataset, the error rate of reverse reads was ∼3 times higher than that of forward reads (*p* = 1.0 × 10^−91^, Wilcoxon signed-rank test). This is likely due to a lower quality of reverse reads, which resulted from oxidation during the sequencing run [20]. Notably, there were some high rate errors in the reverse reads, marked as blue arrows in Fig. 2a. At position 57, the transversion error rate was as high as 12.4 %. In the raw sequencing reads, this position often displayed as ‘N’, which resulted from poor base-calling quality during the sequencing run. After tag-clustering correction, this error was significantly decreased, but was still at 3.4 %. Although our analysis showed that tag-clustering consensus performed better than read-pairing consensus in handling substitution errors, this advantage was not seen in this particular case, which implied the low robustness of tag-clustering method. In conclusion, high quality reads are necessary for avoiding erroneous results from tag-clustering scheme and achieving effective information utilization.

Notably, there were some real mutations in the templates that may arise from potential sources, including mutation accumulation during bacteria clonal formation, PCR procedures, and cross contamination of single mutant samples. Those mutations were buried in the unfiltered dataset but were easily identified after error correction, as indicated by the red arrows in Fig. 2a. The frequencies of real mutations did not change significantly before and after error-correction. This result showed the necessity of error-correction methods for detecting low frequency variants.

### Reproducibility

**Fig. 3:**
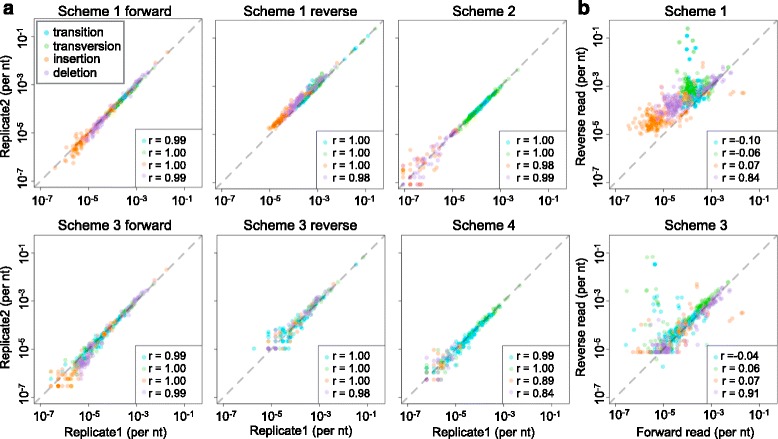
Error reproducibility. **a** The error rate correlation between two technical replicates. Every dot represents a certain position on the target templates. Values on x-axis and y-axis represent error rate at replicate 1 and replicate 2 respectively. **b** The error rate correlation between forward and reverse reads. Every dot represents a certain position on the target templates. Values on x-axis and y-axis represent error rate at forward reads and reverse reads respectively. r is Pearson’s correlation coefficient. The dashed lines are references of complete reproducibility

To confirm the reproducibility of our result, we compared two technical replicates from the same template. All four categories of errors were highly correlated between the technical replicates (Fig. 3a). The high correlation between the error profiles of the raw data implied a sequence-specific error pattern for Illumina sequencing platform [21]. This correlation remained high after error-correction, suggesting that the error-correction methods retained the sequence-specific error patterns.

The prevalence of sequence-specific errors was also evident in the correlation between the forward reads and reverse reads (Fig. 3b). Even for the exact same batch of templates, error patterns between forward reads and reverse reads differed dramatically, as shown by the low correction coefficient. The correlation remained low after tag correction, implying its weakness at correcting sequence-specific errors.

To further examine the error reproducibility, we did a linear regression for the different schemes (Additional file 3: Figure S3). We used the results from the combined consensus to approximate the true mutation rates. According to the previous conclusion, the rates of real mutations remain similar after error-correction, which mapped on the diagonal lines of Additional file 3: Figure S3a. But the sequencing errors were reduced significantly using combined consensus which mapped on the up-left panel of Additional file 3: Figure S3a. Thus, most observed insertions and deletions were due to sequencing errors. However, most observed substitutions comprise both sequencing errors and mutations from the templates.

### Quality score and coverage loss

**Fig. 4:**
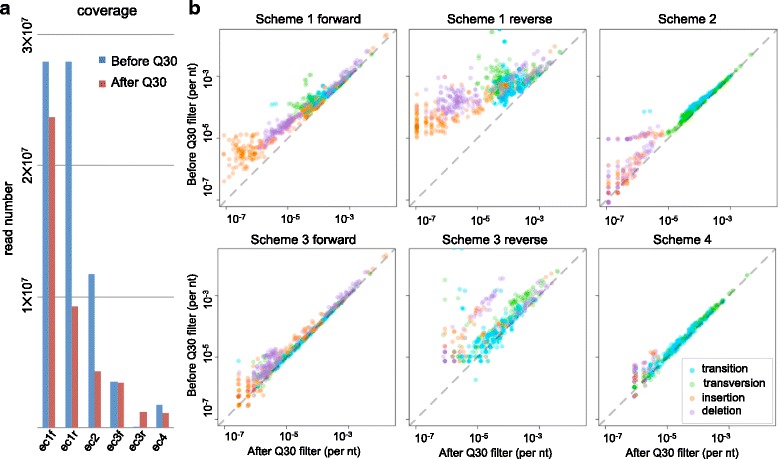
The effect of quality score and coverage. **a** Barplot of coverage in different error-correction schemes, before and after quality score filtering. The labels, ec1f, ec1r, ec2, ec3f, ec3r, and ec4 represent Scheme 1 forward reads, Scheme 1 reverse reads, Scheme 2, Scheme 3 forward reads, Scheme 3 reverse read, Scheme 4, respectively. **b** The errors rate correlation between original data and quality score filtered data. The dashed lines represent complete identical error rates before and after quality score filtering

Coverage loss was one of the major concerns in using the error-correction methods. We counted the read number after each error-correction schemes (Fig. 4a). The coverage of read-pairing correction was 42 % of the raw sequencing data, which was similar to the ideal 50 % loss. Forward reads of tag-clustering correction reached a coverage of 12 % (20 % in the ideal case), while the reverse reads had only 0.4 %. Combined consensus had 6 % coverage of the original data (ideally 10 %). Therefore, our study has shown that using correction methods increases the sequencing cost per nucleotide ∼2.4 fold (1/0.42 ≈ 2.4) for read-pairing correction, ∼8.3 fold (1/0.12 ≈ 8.3) for tag-clustering method (based on forward reads), and ∼17 fold (1/0.06 ≈ 17) for combined consensus. There was a significant trade-off between detection sensitivity and coverage. Researchers needs to consider the balance between coverage loss and detection limit when choosing a suitable error-correction method.

To further analyze the coverage loss of the tag-clustering correction scheme, we examined the cluster size distribution of each unique tag (Additional file 4: Figure S4). In the unfiltered dataset, the cluster size of tags had a bell-shaped distribution if we disregard the high number of single-occurrence tags. The high number of single-occurrence tags was attributed to the sequencing errors at tag regions. With a sequencing error rate of 0.1 % to 1 % at each nucleotide, the chance of having at least one error within the 16-nucleotide tag will be 1.9 % to 10 %. The size for the rest of the read groups distributed smoothly, with a mean of 3.5 for replicate 1, and 4.1 for replicate 2. For the forward reads, where read quality was moderate, tag distribution remained intact after error-correction. For the reverse reads, the number of read groups decreased significantly due to the high inconsistency among sequences within individual read groups. In this study, a given read group would be discarded if the reads within the read group were not completely identical. Therefore, the abundance of low quality reads would cause many read groups being discarded, hence loss of sequencing coverage under tag-clustering correction scheme. In conclusion, the tag-clustering correction method highly depends on precise base-calling at the tag region and high quality reads, which restrict its applications.

Quality score filtering was widely used in error-free sequencing and detecting low frequency variants [15, 22, 23]. We aimed to test the effect of quality score filtering in different analysis schemes. Here we filtered out reads that contained at least one low quality nucleotide (Phred quality score < 30) at the target 88 bp region. In general, the quality score filtering decreased the error rates. Such decrease can be observed by the magnitude of shift of data points to the right of the diagonal line when plotting the error rate before quality score filtering against that of after the quality score filtering (Fig. 4b). Such shift is statistically significant in all analysis schemes in this study (Scheme 1 forward: *p*= 1.4 × 10^−116^, Scheme 1 reverse: *p* = 1.4 ×10^−116^, Scheme 2: *p* = 9.1 ×10^−96^, Scheme 3 forward: *p* = 1.6 ×10^−87^, Scheme 3 reverse: *p*= 4.5 ×10^−59^, and Scheme 4: *p* = 9.9 ×10^−46^, Wilcoxon signed-rank test). Nonetheless, the magnitude of improvement (magnitude of shift of data points to the right of the diagonal line) was generally milder when error-correction schemes were used (Scheme 2, 3, 4). This result indicates that in general, quality score filtering is able to improve the accuracy of read-pairing or tag-clustering error-correction, although the effect is not as dramatic as that of the raw reads.

## Discussion

Over the last decade, next-generation sequencing has become a popular technique in biomedical research due to its increasing throughput and decreasing cost. Illumina sequencing platform is the most widely used next generation sequencing platform, having two shortcomings: high error rate and short read-length. While Illumina has been increasing its read-length through the recent development of MiSeq platform, the error rate remains at ∼0.1 % to 1 % per nt. This error rate may be negligible in certain applications that only require the information of consensus sequence, such as cellular genome sequencing and transcriptome profiling. However, such error rate will significantly impede those applications that require the detection of rare mutations.

Consequently, different experimental approaches have been implemented to overcome this drawback [4–8, 10, 11, 13, 24]. In general, these approaches sacrifice read coverage for a higher sensitivity. Thus, error-correction indirectly increases the per nucleotide cost of sequencing. Therefore, the type of error-correction method should be selected based on the desired sensitivity to minimize the sequencing cost. Here, we proposed several guidelines for choosing an error-correction method, for Illumina HiSeq platform.

1. Error-correction methods should be applied if the required detection limit is lower than 1 %.
2. Read-pairing method is sufficient for detecting variants with frequencies higher than 0.1 %, and is effective for detecting rare insertions and deletions.
3. Tag-clustering method is necessary for detecting variants with frequencies lower than 0.1 %. However, extra depth and high-quality data is needed for carrying out tag-clustering method.
4. Coupling tag-clustering method and read-pairing method is recommended.

We notice that tag-clustering error-correction methods could not avoid certain types of errors. We propose several reasons. Firstly, the sequencing platforms use the first few nucleotides to estimate the parameters for phasing correction. The sequence of tags could induce systematic errors. The templates with the same tags would have the same error in this phasing process [21]. Secondly, the templates with tags were all sequenced at the same time. Thus the buffer quality could result in quality drop at the same position of all reads, which could make tags unable to correct the errors. Thirdly, tags were not amplified or sampled evenly during library preparation. The DNA polymerase had bias for certain primers. In this study, we achieved a polynomial distribution of tags (Additional file 4: Figure S4), which reduced the third systematic error. But tag region itself generated bias.

There are some caveats that limit the power of this study. Firstly, random nucleotide tags were added to the template by PCR. Thus, errors that emerged during the PCR steps cannot be corrected. Such errors should exist here despite a high fidelity DNA polymerase was being used to minimize the PCR errors. The true mutations are therefore comprised of mutations in the original templates (within clone variation), and PCR induced errors. Moreover, there may be cross-contamination from other experiments being performed in the lab that involved mutagenesis. Sampling during plasmid extraction, template amplification, and dilution will also add to the heterogeneity of the templates. In short, the true mutation rate of the sequencing template is not known in this study, which prevents us from precisely quantifying the error rate in each error correction scheme.

While not being addressed in this study, there are numerous computational error-correction methods being developed [25–28]. Most, if not all, of these computational approaches were developed to handle raw sequencing reads. While this study indicates that read filtering based on quality score may only slightly improve the sensitivity, it is unknown whether the sensitivity for deep sequencing may benefit further from combining experimental approach and computational approach. Benchmarking for such integrative error-correction strategy is needed to be done in the future.

Amplicon sequencing is becoming a more popular approach in various research fields because of its high sequencing coverage of a target region of interest. Amplicon sequencing has been widely used in cancer research for diagnosis and disease monitoring purposes [16, 17, 29, 30]. In addition, amplicon sequencing on 16S rDNA gene and other conserved regions is commonly used to characterize the genetic structure of microbe communities [31–33]. Nonetheless, depending on the specific goal, different studies may investigate different genetic regions of interest from different sources of specimens, and employ different sequencing platforms with different read-lengths. In the future, the performance of error-correction strategies should also be evaluated with the consideration of additional parameters, such as samples with extreme GC contents and various degree of genetic diversity, and the usage of other sequencing platforms.

## Methods

### Sequencing library preparation

The target sequence was a synthetic construct of protein G on the pCR-Blunt vector [34] (Additional file 1: Figure S1a). Clonal protein G sequencing template was amplified by PCR using primer pair (replicate 1): 5’-CTA CAC GAC GCT CTT CCG ATC TNN NN A CAN NNN AGT ACG CTA ACG ACA ACG G-3’ and 5’-TGC TGA ACC GCT CTT CCG ATC TNN NNA CAN NNN TCG GAT CCT CCG GAT TCG G-3’, or primer pair (replicate 2): 5’-CTA CAC GAC GCT CTT CCG ATC TNN NN G TGN NNN AGT ACG CTA ACG ACA ACG G-3’ and 5’-TGC TGA ACC GCT CTT CCG ATC TNN NNG TGN NNN TCG GAT CCT CCG GAT TCG G-3’. The underlined nucleotides were served as distinguishing replicate 1 and 2. The eight randomized nucleotides, 4 Ns from each of the forward and reverse primer were served as the tag for error-correction. The entire amplified region (including the primer annealing region) on protein G was 5’-AGT ACG CTA ACG ACA ACG GTG TCG ACG GTG AAT GGA CCT ACG ACG ACG CTA CCA AAA CCT TCA CGG TTA CCG AAT CCG GAG GAT CCG A-3’. The condition of this first PCR was as follow: 2 mins at 95 °C, then 18 three-step cycles of 20 seconds at 95 °C, 15 seconds at 58 °C, and 20 seconds at 68 °C, and a 1 min final extension at 68 °C. The PCR product was purified using PureLink PCR Purification Kit (Life Technologies, Carlsbad, CA). For each sample, ∼6 million copies of the purified PCR product were used for the second PCR. The second PCR was performed using primer pair: 5’-AAT GAT ACG GCG ACC ACC GAG ATC TAC ACT CTT TCC CTA CAC GAC GCT CTT CCG-3’ and 5’-CAA GCA GAA GAC GGC ATA CGA GAT CGG TCT CGG CAT TCC TGC TGA ACC GCT CTT CCG-3’. The condition of the second PCR was the same as that of the first PCR, except 22 cycles were performed instead of 18. All PCRs were performed using KOD DNA polymerase (EMD Millipore, Billerica, MA) with 1.5 mM MgSO4, 0.2 mM of each dNTP (dATP, dCTP, dGTP, and dTTP) and 0.5 *μ*M each of the forward and reverse primers. The resultant product was sequenced by Illumina HiSeq 2500 platform.

### Data analysis

Illumina HiSeq paired-end reads were demultiplexed using the three bp barcode on both forward read and reverse read. The first 12 bp of the read was identified as a tag. For downstream analysis of sequencing error, this 12 bp region was trimmed. As a result, only 88 bp was processed for calculating error rate. After the dataset being processed by the indicated error-correction scheme, pairwise local alignment against the reference protein G sequence was performed. The alignment was carried out using pairwise2 function in the Biopython package [35]. The alignment scoring was as follow: 1 for identical, –1 for mismatching, –1 for gap opening, –0.5 for gap extending. All downstream analyses were performed by custom python scripts.

### Error-correction Scheme 1 (no error-correction)

Errors were called from the raw read. No pairing or quality score filtering was applied on the dataset.

### Error-correction Scheme 2 (read-pairing)

Pairing was performed by comparing the nucleotide sequence of the trimmed foward read and trimmed reverse read (88 bp in both cases). Only those read pairs with a reverse complementary match were used for downstream analysis.

### Error-correction Scheme 3 (tag-clustering)

The tags for the forward read and reverse read were combined and used for grouping reads as described [8]. Briefly, reads that shared the same tag were grouped together as a read group. Read grouping was performed independently for forward read and reverse read. Read groups with a size of less than three reads were discarded. A read group was considered as a real read if all reads in the read group were identical. Otherwise, the read group would be discarded.

### Error-correction Scheme 4 (read-pairing and tag-clustering)

First, read-pairing was performed as described in Scheme 2. The paired reads were then subjected to tag grouping as described in Scheme 3. Of note, under this scheme, read grouping was performed on the paired read instead of independently on forward read and reverse read.

### Availability of supporting data

Raw sequencing data have been submitted to the NIH Short Read Archive (SRA) under accession number: BioProject PRJNA293914. Custom scripts for data analyzing and plotting were deposited in https://github.com/Tian-hao/errorcorrection.

## Additional files

Additional file 1
**Figure S1.** Sequence properties of protein G. (**a**) The sequence of 88 bp template was shown in DRuMS color schemes. The overlapping region of target sequence and forward primer or reverse primer was shown. (**b**) The A-T C-G density plot along the target sequence. Matlab nucleotide sequence analysis toolbox was used to plot this figure. (EPS 498 kb)

Additional file 2
**Figure S2.** Error rates distribution in the original dataset. (**a**) The histogram of error rates. The error rates of four types of errors on every nucleotides were counted. (**b**) Normal Q-Q plot of error rate distribution. Sample quantiles showed great deviation from normal distribution. (EPS 158 kb)

Additional file 3
**Figure S3.** Error rate correlation among different error-correction schemes. (**a**) Linear regression between true mutations and different error-correction methods. The model *y*∼*x*+*a* was adapted to do regression. Every dot represents a position on the target sequence and the values on x-axis and y-axis represent error rates of combined consensus and certain consensus, respectively. Colored lines are regression result. (**b**) Barplot of the intercepts *a* from the linear regression. Error bar is standard error. The colors represents different error-correction schemes, which are labeled in the graph. (EPS 477 kb)

Additional file 4
**Figure S4.** Tag distribution in different error-correction schemes. The histogram of tags. Tags are random nucleotides for readout consensus, comprising 8 nucleotides from each direction of reads. Every bar represents the number of tags that appeared certain times. Scheme 1 means the tag distribution in the original dataset. (EPS 89 kb)

## Abbreviations

NGS: Next-generation sequencing
PCR: Polymerase chain reaction
nt: Nucleotide
Scheme 1: Raw reads
Scheme 2: Read-pairing consensus
Scheme 3: Tag-clustering (Primer ID) consensus
Scheme 4: Combined consensus (read-pairing consensus, followed with tag-clustering consensus)
